# Grain-filling characteristics and yield formation of wheat in two different soil fertility fields in the Huang–Huai–Hai Plain

**DOI:** 10.3389/fpls.2022.932821

**Published:** 2022-07-26

**Authors:** Xuejiao Zheng, Zhenwen Yu, Fengxin Yu, Yu Shi

**Affiliations:** National Key Laboratory of Crop Biology, Agronomy College of Shandong Agricultural University, Tai'an, China

**Keywords:** soil nutrients, yield gap, spike formation, photosynthesis characteristics, grain filling

## Abstract

Clarifying factors that underpinning the variation in wheat yield components between high and middle soil fertility fields is critical to increase grain production and narrow yield gap for smallholder farming systems in the Huang–Huai–Hai Plain (3HP), which characterized by a large variation in soil fertility. Two-year field experiments were conducted to investigate wheat tillering, leaf photosynthesis, and grain filling characteristics in different soil fertility fields: high soil fertility field (HF) and middle soil fertility field (MF). Results showed that the spike formation rate in HF was 12.7%–13.0% higher than that in MF, leading to an 18.0%–19.8% increase in spike number. In addition, HF improved canopy light interception and leaf photosynthesis characteristics after anthesis and delayed leaf senescence, contributing to the increase in both the active grain filling period and grain filling rate. This resulted in a higher 1,000 grain weight in HF, which was 8.2%–8.3% higher than that in MF. Compared to MF, HF obtained higher yields at 9,840 kg ha^−1^ in 2017/18 and 11,462 kg ha^−1^ in 2018/19, respectively. In summary, higher spike number and 1,000-grain weight, which were mediated by spike-formation rate, maximization of light interception and improved leaf photosynthesis. These results would have important implications for narrowing yield gap between MF and HF in the 3HP.

## Introduction

Food production is a great challenge in China for its limited farmland resources and large population of more than 1.40 billion citizens need to be fed (Lu and Fan, 2013; National Bureau of Statistics of China, 2020). The Huang–Huai–Hai Plain (3HP) is the primary winter wheat production region in China, producing approximately 61% of the wheat grown in China (Xu et al., 2018). In this region, the mean yield potential for winter wheat varied between 5,000 and 8,000 kg ha^−1^ from 1981 to 2008 (Chen et al., 2017), with the most recent attainable potential yield at 12,611 kg ha^−1^ between 2018 and 2019 (Farmer’s Daily, 2019).^1^ This yield gap can be attributed to irrigation, high-yield wheat cultivars, and different gradients of soil fertility in croplands arising from fertilization replenishment (Xiao and Tao, 2014; Chen et al., 2017; Wang et al., 2018). Smallholder fields in the 3HP have played a dominant role in crop production for decades (Feng et al., 2020), and these farming systems are characterized by a large variation in soil fertility (Tittonell et al., 2007). To increase wheat production and narrow the yield gap, studies on the physiological characteristics of wheat grown in a high soil fertility field (HF) with high potential yield are pivotal.

Soil fertility is defined as the quality of a soil to supply nutrients in adequate amounts and proper balance for the growth of crop plants (Fageria, 2007). Hag Husein et al. (2021) indicated that soil fertility increases as the soil organic matter (SOM) content increases. Soil nutrient supply and nutrient cycling in different soil fertility fields substantially affect crop growth and grain yield (Kautz et al., 2013; Zhang et al., 2018, 2019a). Wang et al. (2018) reported that nitrogen (N) contributes to the maintenance of SOM, and SOM at 10.0–14.9 g kg^−1^, total nitrogen (TN) at 1.0–1.5 g kg^−1^, available phosphorus (AP) at 10.0–19.8 mg kg^−1^, and available potassium (AK) at 100–200 mg kg^−1^ were the most effective soil fertility levels to improve wheat grain yield. Soil potassium levels had significant effect in tillering, spike number, and yield of wheat (Abbas et al., 2013). The application of maize straw mulch combined with no-tillage could increase plant-available soil N, thus promoting wheat tillering and grain yield (Yang et al., 2020). When water availability was not limited for wheat production, increased soil N arising from increased N application improved the tiller appearance rate, thereby leading to an increase in the maximum tiller number (Alzueta et al., 2012). Rational populations ensure superior canopy light interception and penetration and improve the light utilization efficiency of plants (Hussain et al., 2013). There have been many studies on the response of wheat tillering generation to different soil fertility fields, but most of these have focused on yield levels ranging from 4,500 to 9,000 kg ha^−1^ (Allard et al., 2013; Yang et al., 2020). However, there is a knowledge gap regarding the changes in soil nutrient content and tillering of winter wheat in fields with grain yield levels ranging from 9,000 to 11,000 kg ha^−1^.

Moreover, increased photosynthetic capacity and delayed leaf senescence are especially important for increasing crop yields (Wang et al., 2020). It is well known that flag-leaf photosynthesis contributes approximately 30%–50% of the assimilation for grain filling (Guo et al., 2015), whereas leaf photosynthetic performance would inevitably decline during grain filling because the initiation of grain filling coincides with the onset of senescence and the photosynthetic apparatus gradually breaks down (Man et al., 2017). Different soil nutrient status arising from tillage methods influenced the photosynthetic capacity and aging process of wheat, thereby grain filling (He et al., 2020). HF with grain-yield levels up to 11,000 kg ha^−1^ provides an excellent opportunity to identify physiological features associated with the attainable potential yield of wheat; therefore, it is necessary to study leaf photosynthesis and senescence and grain filling characteristics during the grain filling stage in HF.

This study aimed to investigate the differences in soil nutrient content, wheat tillering, light interception characteristics, photosynthesis and the senescence of flag leaves and grain filling characteristics of two different soil fertility fields with different grain-yield levels. This can be helpful to clarify the variation of yield components in different soil fertility fields and provide useful information for high-yielding wheat production.

## Materials and methods

### Experimental site description

**Figure 1:**
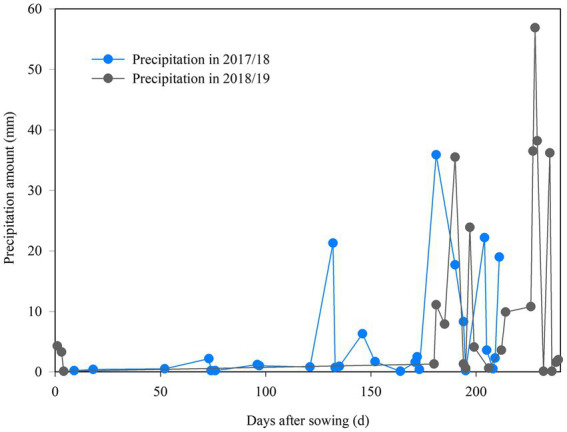
Daily precipitation during the winter wheat-growing seasons in 2017/18 and 2018/19.

Field experiments were conducted at Shijiawangzi village (116°41′E, 35°42’N), located in Yanzhou, Shandong Province, China. This area has a temperate, continental monsoon climate with an annual average temperature of 13.6°C, an annual cumulative sunshine hour of 2,460.9 h, an annual rainfall of 621.2 mm, of which 40% is received during the winter wheat-growing season (October–June). The rotation of winter wheat and summer maize is the main cropping system in this region. Daily precipitation during the wheat-growing season in 2017/18 and 2018/19 are shown in Figure 1.

### Experimental design

High soil fertility wheat field (with grain-yield levels up to 11,000 kg ha^−1^) and middle soil fertility wheat field (with grain-yield levels up to 9,000 kg ha^−1^) were selected and represented by HF and MF, respectively. HF and MF are flat area and belong to two farmers. The straight-line distance between HF and MF is 100 m. The soil type of HF and MF is Haplic luvisol (FAO classification system). Before wheat sowing, additional 3,750 kg (0.65%N, 0.23% P_2_O_5_ and 0.78% K_2_O content) organic fertilizer was applied regularly in HF from 2001 to 2016, but the organic fertilizer was applied in MF only in 2016. The HF and MF with wheat yields of 11,015 and 9,336 kg ha^−1^, respectively, in 2016/17, and the yield gap between HF and MF was mainly caused by soil fertility (Yu et al., 2018). Annual precipitation were the same in HF and MF. The soil texture, bulk density, and field capacity at the experimental site before sowing are shown in Supplementary Tables S1 and S2. The soil particle size distribution was analyzed using the hydrometer method, and the triangle coordinates of the American system of soil texture classification was used to determine the soil texture. Based on the analysis of soil samples collected from the experimental site in 2017 and 2018 before sowing, the initial amount of soil nutrients (0–20 cm soil layer) in the two fields are shown in Table 1.

**Table 1:** Soil nutrient conditions in the 0–20 and 20–40 cm soil layers under different treatments before sowing, at jointing, and anthesis stages in 2017/18 and 2018/19 growing seasons.

Year	Stages	Treatments	Organic matter (g kg^−1^)		Total nitrogen (g kg^−1^)		Hydrolyzable nitrogen (mg kg^−1^)		Available phosphorus (mg kg^−1^)		Available potassium (mg kg^−1^)	
			0–20	20–40	0–20	20–40	0–20	20–40	0–20	20–40	0–20	20–40
2017/18	Before sowing	HF	19.20a	9.52a	1.20a	0.60a	166.30a	105.05a	56.20a	41.56a	204.29a	169.33a
		MF	15.02b	8.32b	1.12b	0.51b	116.50b	93.33b	35.13b	26.32b	118.15b	86.38b
	Jointing	HF	19.42a	9.71a	1.27a	0.67a	170.44a	112.46a	59.88a	44.32a	211.07a	175.39a
		MF	15.19b	8.51b	1.18b	0.56b	119.49b	97.90b	37.64b	28.72b	124.52b	92.02b
	Anthesis	HF	19.28a	9.55a	1.23a	0.64a	168.64a	110.99a	57.67a	42.21a	206.83a	171.76a
		MF	15.08b	8.39b	1.17b	0.55b	118.36b	96.66b	36.40b	27.45b	122.13b	88.65b
2018/19	Before sowing	HF	19.28a	9.57a	1.21a	0.62a	168.21a	105.41a	57.77a	42.70a	206.01a	170.79a
		MF	15.11b	8.35b	1.13b	0.50b	117.74b	95.06b	36.29b	26.66b	119.19b	87.18b
	Jointing	HF	19.51a	9.75a	1.28a	0.68a	171.07a	112.84a	61.45a	45.18a	211.10a	176.50a
		MF	15.31b	8.51b	1.20b	0.56b	119.17b	98.60b	38.18b	28.65b	124.16b	91.39b
	Anthesis	HF	19.32a	9.63a	1.24a	0.65a	169.64a	112.20a	58.58a	43.11a	208.62a	174.14a
		MF	15.20b	8.46b	1.18b	0.55b	118.89b	98.06b	36.60b	28.11b	123.67b	89.98b

HF, high soil fertility field and MF, middle soil fertility field. Values followed by a different letter are significantly different (LSD test, *p* < 0.05) within the treatments in each year.

Seeds of wheat cultivar Yannong 1212 (broken the yield record of winter wheat twice in China since 2015 and showed high yields of over 12,000 kg ha^−1^) (Dazhong Daily, 2020)^2^ were planted in HF and MF. The plot size was 60 m^2^ (30 m long × 2 m wide) with three replicates and had a 2.0-m wide isolation area setting between each plot to minimize the effects of adjacent plots. The straw of the preceding maize crop was crushed and returned to the soil. Base fertilizer of 105 kg ha^−1^ N, 150 kg ha^−1^ P_2_O_5_, and 150 kg ha^−1^ K_2_O were surface-applied to the soil before sowing. At the jointing stage, each plot was fertilized with 165 kg ha^−1^ N by ditching. Fertilizer types were urea (46.4% N content), diammonium phosphate (46% P_2_O_5_ and 18% N content), and potassium sulfate (52% K_2_O content). Wheat seeds were sown on 23 October 2017, and 10 October 2018, with a population of 270 plants m^−2^ and 180 plants m^−2^, respectively. Wheat plants were harvested on 7 June 2018 and 11 June 2019. Other crop management was the same as used in local wheat production.

### Sampling and measurements

#### Soil nutrient contents

Soil samples from 0 to 20 and 20 to 40 cm soil layers were randomly collected using a soil corer from each plot with three replicates before sowing, 1 day before irrigation at jointing and anthesis. SOM was determined using the K_2_Cr_2_O_7_-H_2_SO_4_ oxidation method, and TN was determined using the semimicro-Kjeldahl method (He et al., 2019). Soil hydrolyzable N, AP, and AK were measured using the routine soil sample laboratory analytical method described by Bao (2000).

### Tillering production and survival

The number of maximum tillers at re-greening was determined by counting all the stems from three random sample areas of 5 m^2^ in each plot. The spike number at maturity was estimated by counting all spikes from the harvested area of 5 m^2^ in each plot. The tillering capacity (no. plant^−1^) and spike formation rate (%) were calculated using the equation described by Yang et al. (2020):


$$\begin{array}{c} \mathrm{Tillering capacity}=\left(\mathrm{maximum tillers}-\mathrm{basic seedlings}\right)\\ /\mathrm{basic seedlings}\times 100 \end{array}$$

Spike formation rate=spike number/maximum tillers×100

### Photosynthetically active radiation measurements

Intercepted photosynthetically active radiation (PAR_i_) was measured using the SS1 SunScan canopy analysis system (Delta-T Devices, Cambridge, United Kingdom) in three randomly non-destructive sampling areas from each plot between 10:00 am and 11:30 am at 7-day intervals from anthesis to 35 days after anthesis (DAA). With the reference photosynthetically active radiation (PAR) sunshine sensor, the incident PAR was measuring above and beneath wheat canopy by the 1-m probe. The fraction of PAR_i_ (PARF, %) was estimated according to the method described by Davidenco et al. (2020) and Liu et al. (2020):


$$\mathrm{PARF}=\left[1-\left({\mathrm{PAR}}_{0}/{\mathrm{PAR}}_{t}\right)\right]\times 100$$

where PAR_0_ (μmol m^−1^ s^−1^) is the incident PAR at the surface of the ground and PAR_t_ (μmol m^−1^ s^−1^) is the incident PAR at 50 cm above the wheat canopy.

### Gas exchange parameters of flag leaves

Flag-leaf gas exchange parameters, including net photosynthetic rate (Pn) and transpiration rate (Tr), were measured using a LI-6400XT portable gas exchange measuring system (LI-COR, Lincoln, Nebraska, United States). Measurements were taken with three representative flag leaves from each plot between 9:00 am and 11:00 am at 7-day intervals from anthesis to 35 DAA, under the conditions of 25°C, 500 μmol s^−1^ flow rate, atmospheric CO_2_ concentration, and 1,300 mol m^−2^ s^−1^ photosynthetic active radiation in a 2 × 3 cm^2^ leaf chamber. The instantaneous water use efficiency (WUE_Leaf_) of flag leaves was calculated using the equation (Fan et al., 2019):


$${\mathrm{WUE}}_{\mathrm{Leaf}}=\mathrm{Pn}/\mathrm{Tr}$$

where WUE_Leaf_ (μmol CO_2_/mmol H_2_O) is the instantaneous water use efficiency of flag leaves, Pn (μmol CO_2_ m^−2^ s^−1^) is the net photosynthetic rate of flag leaves, and Tr (mmol H_2_O m^−2^ s^−1^) is the transpiration rate of flag leaves.

### Antioxidant enzyme activities of flag leaves

Twenty representative flag leaves from each plot were sampled at 7-day intervals from anthesis to 28 DAA. Fresh samples were immediately submerged in liquid N after collection and stored at −80°C for enzyme assays. Malondialdehyde (MDA) concentration, superoxide dismutase (SOD) activity, and soluble protein content were assayed following the method described by Guo et al. (2015).

### Grain filling traits

Spikes emerging on the same day were chosen and tagged in each plot. Twenty spikes were collected from the tagged samples at 7-day intervals from anthesis to 35 DAA. After each sampling, spikes were heated at 105°C for 10 min, dried at 70°C to a constant weight, threshed, hand hulled, and counted before recording the grain weight. Grain filling was fitted using a logistic function (Li et al., 2020):


$$W=A/\left(1+{\mathrm{Be}}^{-C\times t}\right)$$

where W (g) is the 1,000-grain dry weight at time t, A (g) is the theoretical final dry weight of 1,000-grain, t (d) is the day after anthesis, and B and C are the coefficients determined by regression.

The following values, including the duration of grain filling (T), 1,000-grain weight at maximum grain-filling rate (W_max_), maximum grain-filling rate (V_max_), mean grain-filling rate (V_mean_), and active grain-filling period (D) were calculated according to the method proposed by He et al. (2020):


T=(lnB+4.59512)/C

$$W_{\max}=A/2$$

$$V_{\max}=\left(C\times W_{\max}\right)\times \left(1-W_{\max}/A\right)$$

$$V_{\mathrm{mean}}=A/\left(1+{\mathrm{Be}}^{-C\times T}\right)/T$$

D=6/C

### Grain Yield and Yield Components

All plants were harvested in an area of 5 m^2^ from each plot at maturity, threshed, sun-dried to 12.5% moisture content, and weighed to calculate the grain yield. Total spikes of each sampled area were counted to determined the spike number. Twenty plants were selected randomly to recorded the number of grains per spike in each sampled area. 1,000-grain weight was determined by weighing 1,000 grains from the yield measurement samples in each sampled area with three replicates.

### Statistical analysis

Data computation and statistical analyses were performed using Microsoft Excel 2016 and SPSS 22.0 (IBM Corp. Released 2013. IBM SPSS Statistics for Windows, Version 22.0. Armonk, NY: IBM Corp.). Treatment means were tested using the least significant difference (LSD) test at a 5% level of significance (*p* < 0.05). Pearson’s correlation analyses were used to reveal the relationships between soil nutrients, yield components and grain yield, and the relationships between PARF, photosynthesis and senescence characteristics of flag leaves after anthesis, grain filling characteristics and grain yield in a combined analysis of data over 2 years.

## Results

### Soil nutrient conditions

Table 1 shows the soil nutrient conditions under different treatments before sowing, at jointing and anthesis stages. In both years, HF showed higher SOM in the 0–20 cm soil layer than MF before sowing, at jointing and anthesis stages. In the 0–20 cm soil layer, TN in HF was 7.1%, 7.6%, and 5.1%, before sowing, at jointing and anthesis, higher than in MF in 2017/18, respectively. Compared to MF, HF showed higher hydrolyzable N, AP, and AK before sowing, at jointing and anthesis in 2017/18 and 2018/19. In the 20–40 cm soil layer, soil nutrient conditions in HF were higher than MF before sowing, at the jointing and anthesis stages in both years.

### Tillering production and survival

**Figure 2:**
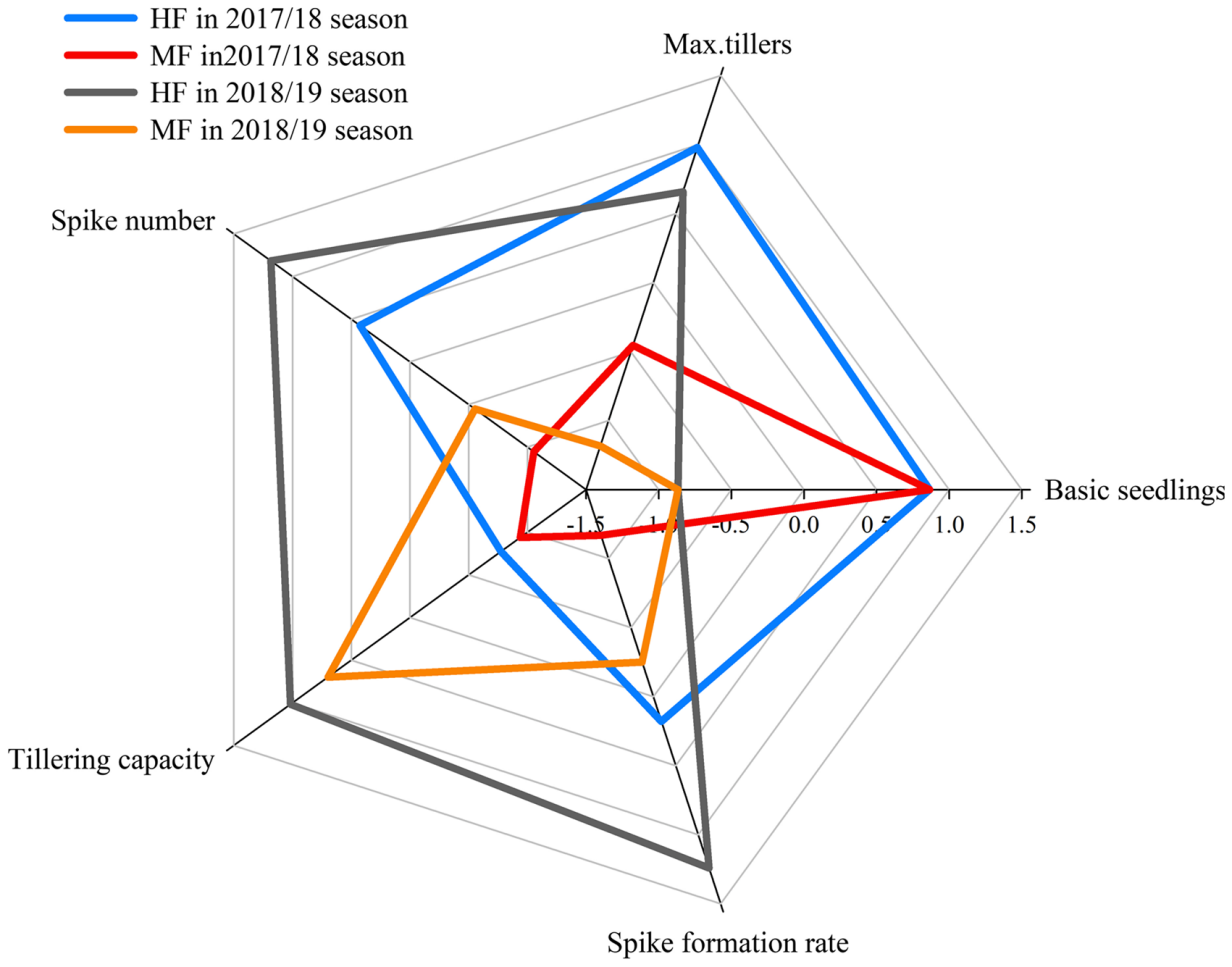
Comprehensive performance of basic seedlings, maximum tillers, spike number, tillering capacity, and spike formation rate of wheat in 2017/18 and 2018/19. HF, high soil fertility field and MF, middle soil fertility field. All data were standardized.

The maximum tillers of wheat in HF were increased by 4.7% and 6.2% in 2017/18 and 2018/19, respectively, compared to those for MF (Table 2). HF showed greater spike number and tillering capacity than MF in both years. Compared to MF, HF significantly improved the spike formation rate with increases of 12.7% and 13.0% in 2017/18 and 2018/19, respectively. The comprehensive analysis of the indexes above showed that despite the difference in basic seedlings in 2017/18 and 2018/19, the order of spike number from high to low among the treatments and years was consistent with the spike formation rate (Figure 2).

**Table 2:** Effects of soil fertility on tillering and spike-forming characteristics of wheat in 2017/18 and 2018/19 growing seasons.

Year	Treatments	Basic seedlings (×10^4^ ha^−1^)	Maximum tillers (×10^4^ ha^−1^)	Spike number (×10^4^ ha^−1^)	Tillering capacity (no. plant^−1^)	Spike formation rate (%)
2017/18	HF	270	1871a	728a	5.93a	38.93a
	MF	270	1787b	617b	5.62b	34.53b
2018/19	HF	180	1852a	785a	9.29a	42.40a
	MF	180	1744b	655b	8.69b	37.53b

HF, high soil fertility field and MF, middle soil fertility field. Values followed by a different letter are significantly different (LSD test, *p* < 0.05) within the treatments in each year.

### PAR capture

**Figure 3:**
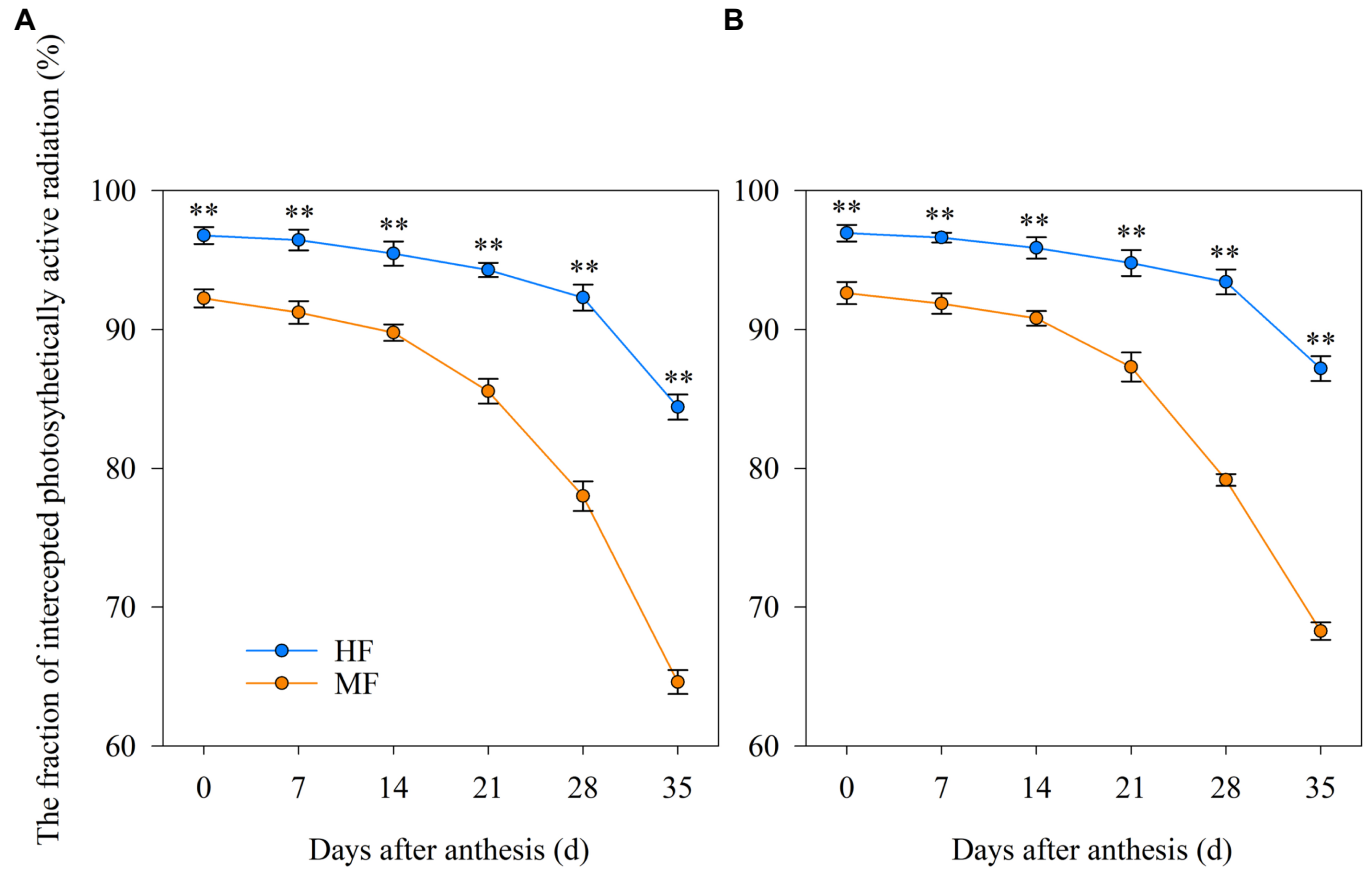
Dynamics of the intercepted photosynthetically active radiation fraction of wheat after anthesis in 2017/18 **(A)** and 2018/19 **(B)**. HF, high soil fertility field and MF, middle soil fertility field. Vertical bars represent the SD of the means. **p < 0.01.

According to the results shown by Figure 3, soil fertility affected the PAR capture of wheat after anthesis. Compared to MF, HF significantly improved PARF after anthesis in both years. The effects were significantly enhanced from 21 DAA, and the PARF at 35 DAA in HF increased by 30.7% and 27.7% in 2017/18 and 2018/19, respectively, compared to MF.

### Photosynthesis characteristics after anthesis

**Figure 4:**
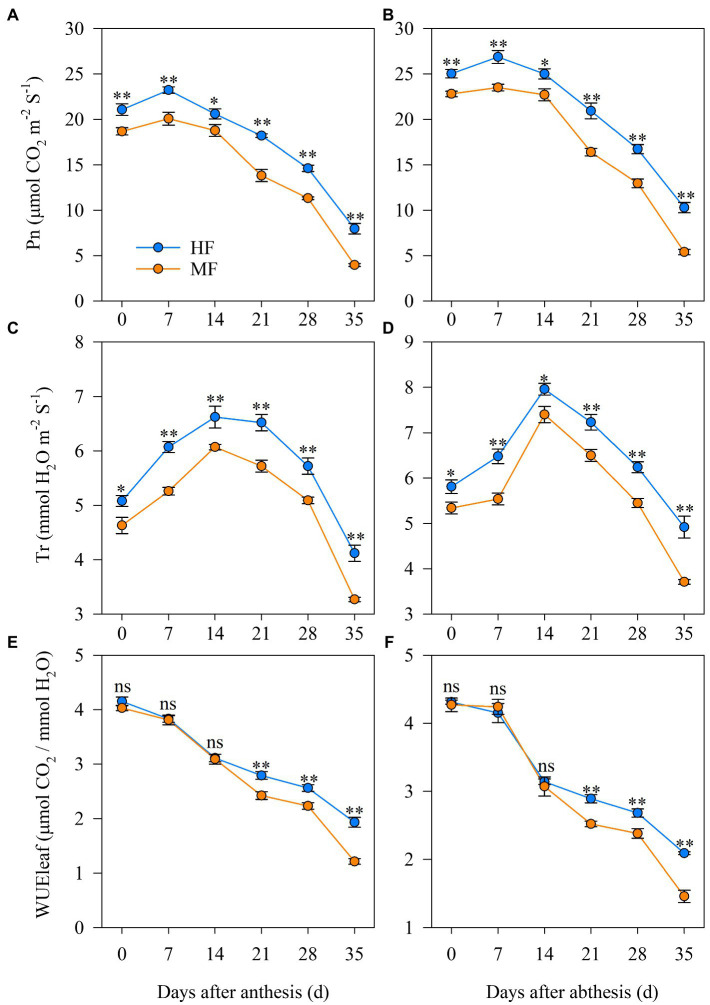
Dynamics of the photosynthesis characteristics of flag leaves after anthesis in 2017/18 **(A,C,E)** and 2018/19 **(B,D,F)** growing seasons. HF, high soil fertility field and MF, middle soil fertility field. Vertical bars represent the SD of the means. ns, *p* > 0.05; *p < 0.05 and **p < 0.01.

Figure 4 shows the differences in photosynthesis characteristics after anthesis between HF and MF. In 2017/18, the Pn at 0, 7, 14, 21, 28, and 35 DAA in HF increased by 12.9%, 15.7%, 9.7%, 31.6%, 29.1%, and 100.8%, respectively, compared to MF. Moreover, MF significantly improved Tr from 0 to 35 DAA. Similar trends were observed in 2018/19. However, there was no considerable difference in WUE_Leaf_ at 0, 7, and 14 DAA between HF and MF. HF showed higher WUE_Leaf_ at 21, 28, and 35 DAA than those of MF in both years.

### Leaf senescence characteristics after anthesis

**Figure 5:**
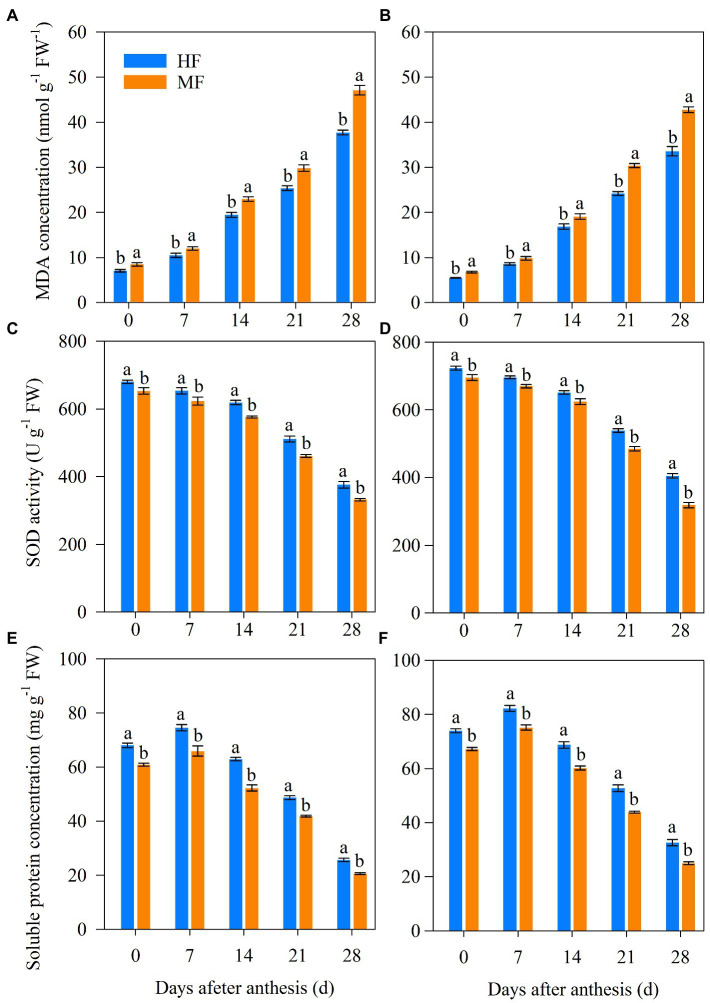
Malondialdehyde (MDA) concentration, superoxide dismutase (SOD) activity, and soluble protein (SP) concentration of flag leaves after anthesis in 2017/18 **(A,C,E)** and 2018/19 **(B,D,F)** growing seasons. HF, high soil fertility field and MF, middle soil fertility field. Vertical bars represent SD of the means. Different letters indicate statistical significance at *p* < 0.05 among treatments.

According to the results shown in Figure 5, HF decreased the MDA concentration from 0 to 35 DAA compared to MF in both years contributing to the delayed leaf senescence. In addition, the SOD activity of flag leaves at 0, 7, 14, 21, and 28 DAA in HF were 4.1%, 4.9%, 7.5%, 10.9%, and 13.2%, respectively, higher than those of MF in 2017/18, respectively. Similar trends were observed in 2018/19. The SP concentration of flag leaves increased and then decreased after anthesis in both years. SP concentrations at 0, 7, 14, 21, and 28 DAA were higher for HF than MF.

### Grain filling characteristics

**Figure 6:**
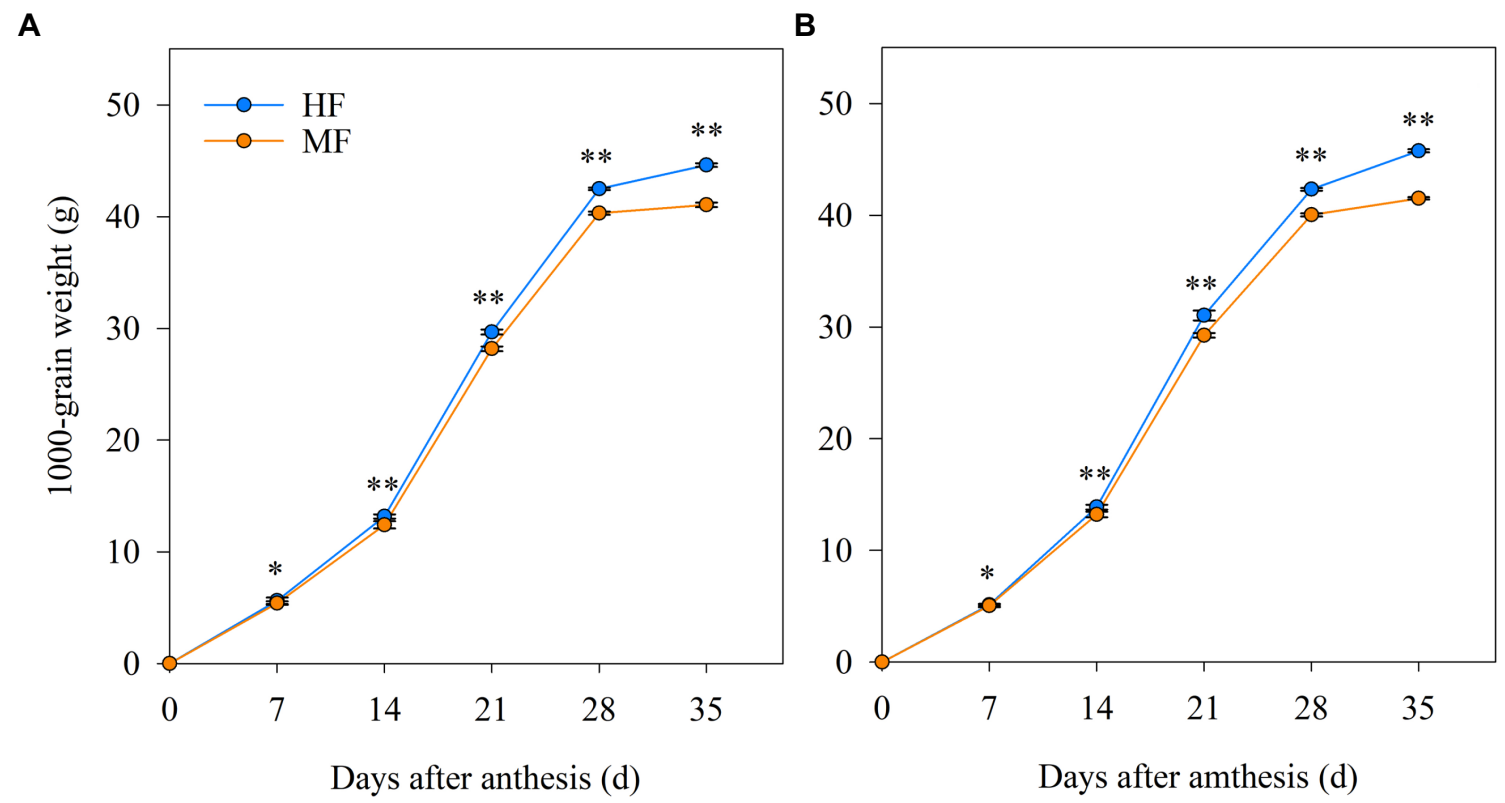
The 1,000-grain weight after anthesis in 2017/18 **(A)** and 2018/19 **(B)** growing seasons. HF, high soil fertility field and MF, middle soil fertility field. Vertical bars represent SD of the means. *p < 0.05 and **p < 0.01.

The 1,000-grain weight after anthesis is shown in Figure 6. The increase rate of the 1,000-grain weight was enhanced from 14 to 21 DAA and 21 to 28 DAA than from 0 to 7, 7 to 14, and 28 to 35 DAA. Moreover, HF significantly increased the 1,000-grain weight at 7, 14, 21, 28, and 35 DAA compared to MF. Table 3 shows that the grain filling processes in HF and MF were both fitted to the logistic growth curve equation in 2017/18 (correlation coefficient ≥ 0.9976) and 2018/19 (correlation coefficient ≥ 0.9989). Compared to MF, the W_max_ in HF increased by 8.3% and 9.5% in 2017/18 and 2018/19, respectively. The V_max_ and V_mean_ of HF were 4.7% and 5.5% higher than those of MF in 2017/2018 and 5.9% and 6.3% higher in 2018/19, respectively. D in HF was longer than that in MF in both years. Thus, compared to MF, the 1,000-grain weight superiority after anthesis in HF was due to its higher grain filling rate and longer active filling period.

**Table 3:** The grain filling process model and grain filling parameters in 2017/18 and 2018/19 growing seasons.

Year	Treatment	Growth curve equation	Correlation coefficient	W_max_ (mg grain^−1^)	V_max_ (mg grain^−1^ d^−1^)	V_mean_ (mg grain^−1^ d^−1^)	D (d)
2017/18	HF	*y* = 46.6699/(1 + 45.4756e^-0.2099x^)	0.9985	23.33a	2.45a	1.15a	28.59a
	MF	*y* = 43.0980/(1 + 48.5304e^-0.2173x^)	0.9976	21.55b	2.34b	1.09b	27.61b
2018/19	HF	*y* = 47.1049/(1 + 45.5225e^-0.2129x^)	0.9995	23.55a	2.51a	1.18a	28.18a
	MF	y = 43.0060/(1 + 47.3994e^-0.2207x^)	0.9989	21.50b	2.37b	1.11b	27.18b

W_max_, weight of maximum grain filling rate; V_max_, maximum grain-filling rate; V_mean_, mean grain-filling rate; D, active grain filling period; HF, high soil fertility field; and MF, middle soil fertility field. Values followed by a different letter are significantly different (LSD test, *p* < 0.05) within the treatments in each year.

### Grain yield and yield components

Compared to MF, HF significantly improved the spike number and 1,000-grain weight, which resulted in an increase in grain yield and compensated for the decrease in grain number (Table 4). The grain yield was 20.4% and 24.1% greater in HF than in MF in 2017/18 and 2018/19, respectively.

**Table 4:** Yield components and grain yield under different treatments in 2017/18 and 2018/19 growing seasons.

Year	Treatment	Spike number (×10^4^ ha^−1^)	Grain number per spike	1,000-grain weight (g)	Grain yield (kg ha^−1^)
2017/18	HF	728a	36.58b	44.57a	9840a
	MF	617b	39.08a	41.14b	8176b
2018/19	HF	785a	38.85b	45.37a	11462a
	MF	655b	40.72a	41.92b	9239b

HF, high soil fertility field and MF, middle soil fertility field. Values followed by a different letter are significantly different (LSD test, *p* < 0.05) within the treatments in each year.

### Correlation analysis

Correlation analysis showed that mean soil nutrients at 0–40 cm (SOM, TN, hydrolyzable N, AP and AK) during whole wheat growth stages were positively correlated with spike number, 1,000-grain weight, and grain yield (Table 5). Grain number had a negative relationship with mean soil nutrients at 0–40 cm during the whole wheat growth stages. Moreover, grain filling characteristic parameters (W_max_, V_max_, and V_mean_) and grain yield were positively correlated with PARF, photosynthetic characteristics (Pn, Tr and WUE_Leaf_) and senescence characteristics (SOD and SP), and were negatively correlated with MDA (Table 6). D was positively correlated to the PARF.

**Table 5:** Analysis of correlations among mean soil nutrients in 0–40 cm soil layer during whole wheat growth stages, yield components, and grain yield in 2017/18 and 2018/19 (*n* = 12).

	SOM	TN	HN	AP	AK	SN	GN	GW	GY
SOM	1								
TN	0.970^**^	1							
HN	0.999^**^	0.972^**^	1						
AP	0.998^**^	0.975^**^	0.999^**^	1					
AK	0.999^**^	0.973^**^	1.000^**^	0.999^**^	1				
SN	0.928^**^	0.918^**^	0.929^**^	0.933^**^	0.924^**^	1			
GN	−0.683^*^	−0.657^*^	−0.685^*^	−0.671^*^	−0.692^*^	−0.402	1		
GW	0.969^**^	0.957^**^	0.969^**^	0.970^**^	0.966^**^	0.966^**^	−0.540	1	
GY	0.830^**^	0.831^**^	0.832^**^	0.841^**^	0.823^**^	0.964^**^	−0.212	0.911^**^	1

SOM, organic matter; TN, total nitrogen; HN, hydrolyzable nitrogen; AP, available phosphorus; AK, available potassium; SN, spike number; GN, grain number; GW, 1,000-grain weight; and GY, grain yield.

* Correlation is significant at the 0.05 level (2-tailed).

** Correlation is significant at the 0.01 level (2-tailed).

**Table 6:** Analysis of correlations among intercepted photosynthetically active radiation fraction (PARF), photosynthesis and senescence characteristics of flag leaves after anthesis, grain filling characteristic parameters, grain yield (GY) in 2017/18 and 2018/19 (*n*  = 12).

	PARF	Pn	Tr	WUE_Leaf_	MDA	SOD	SP
W_max_	0.989^**^	0.768^**^	0.750^**^	0.805^**^	−0.883^**^	0.814^**^	0.809^**^
V_max_	0.939^**^	0.896^**^	0.900^**^	0.859^**^	−0.941^**^	0.914^**^	0.908^**^
V_mean_	0.946^**^	0.920^**^	0.922^**^	0.898^**^	−0.965^**^	0.936^**^	0.935^**^
D	0.818^**^	0.408	0.365	0.535	−0.592^*^	0.481	0.481
GY	0.873^**^	0.984^**^	0.977^**^	0.961^**^	−0.986^**^	0.994^**^	0.990^**^

Pn, net photosynthetic rate; Tr, transpiration rate; WUE_Leaf_, instantaneous water use efficiency; MDA, malondialdehyde concentration; SOD, superoxide dismutase activity; SP, soluble protein concentration; W_max_, weight of maximum grain filling rate; V_max_, maximum grain-filling rate; V_mean_, mean grain-filling rate; and D, active grain filling period.

* Correlation is significant at the 0.05 level (2-tailed).

** Correlation is significant at the 0.01 level (2-tailed).

## Discussion

The composition of tillers directly affects the structure and quality of the wheat population, which is highly related to grain yield (Cai et al., 2014). Shang et al. (2021) indicated that N regulates tiller bud growth by regulating N metabolism and can be redistributed from the senescent and aborted tillers to the rest of plant to improve tiller survival. When yield levels ranging from 4,500 to 6,000 kg ha^−1^, low N rates (210 kg ha^−1^ vs. 240 and 270 kg ha^−1^) had a prominent decrease in spike formation rate, resulting in a decreased spike number at maturity (Ding et al., 2021). However, Wang et al. (2021) found that N application alone had less effect on wheat tiller number, while N and phosphorus combination can significantly increase the tiller number. In our results, soil nutrients in the 0–40 cm soil layers were higher for HF (with grain yield levels up to 11,000 kg ha^−1^) than for MF (with grain yield levels up to 9,000 kg ha^−1^) during the wheat growth period from 2017 to 2019 (Table 1). This ensured the soil N and phosphorus requirements for the wheat tillering in HF. In this study, the significantly increases of maximum tiller number, tillering capacity and spike formation rate shown in HF compared to MF (Table 2) can be attributed to the improved soil nutrients, since soil nutrients can regulate wheat tillering (Otteson et al., 2008; Wang et al., 2021). Although the basic seedlings in 2017/18 and 2018/19 were different, the increase of spike number was consistent with the increase of spike formation rate among the treatments and years in the comprehensive analysis (Figure 2). These results suggested that the spike formation rate of wheat contributed most to the spike number rather than the maximum tiller number or tillering capacity. This result agrees with that of Hu et al. (2017), who reported that increasing the spike formation rate reduced growth competition and wastage of photosynthates from ineffective tillers, which is an effective way to increase productive tiller number.

Canopy light interception plays a crucial role in elevating crop productivity (Plénet et al., 2000; Zhang et al., 2019b). Bingham et al. (2019) indicated that the protection of canopy PAR interception was required to maximize grain yield until approximately 80% of the grain filling was completed in spring barley. With yield levels ranging from 3,300 to 4,800 kg ha^−1^, PARF in the middle and lower layers of wheat canopy can be increased by applying N fertilizer ranging from 90 to 180 kg ha^−1^, but excessive N application at 270 kg ha^−1^ led to the decreased canopy PARF (Luo et al., 2021). Inferior soil N availability due to the decreased N application rates resulted in a decrease in the PARF of crops, which could be attributed to the decreased leaf area index (Hamzei and Soltani, 2012). In this study, different soil fertility fields affected the PARF, and HF increased PARF after anthesis by 4.7%–30.7% as compared to MF (Figure 3), which ensured energy availability for photosynthesis during the grain filling stage. This result could be attributed to the higher soil nutrients for HF during the wheat growth period (Table 1), as different N fertilizer amounts has a great impact on PARF (Kandel et al., 2019). Moreover, positive relationships were observed among PARF and grain filling characteristic parameters (Table 6), suggesting that improved canopy light interception was beneficial for the increasing of the grain filling rate and for the extension of grain filling duration.

Photosynthesis is the main driver of biomass accumulation and yield (Parry et al., 2011) and is highly sensitive to soil N deficit (Zhang et al., 2017). The decrease in Pn is due to the start of leaf senescence, and the photosynthetic machinery rapidly disassembles, reducing the photosynthetic capacity of flag leaves (Brouwer et al., 2012; Man et al., 2017). The disturbance of soil nutrients affected by tillage influenced leaf senescence in wheat (He et al., 2020). Comparable results were found in our study where MF with grain yield levels up to 9,000 kg ha^−1^ had lower soil nutrients leading to earlier leaf senescence after anthesis as compared to HF (Table 1; Figure 5). In HF, the higher SOD activity and the SP concentration of flag leaves relieved the damage of the leaf protective enzyme system (Figure 5) and increased the photosynthetic characteristics (Figure 4), lengthening the duration of leaf photosynthesis during grain filling. Moreover, positive relationships between deferred senescence of flag leaves and grain yield were found in this study (Table 6) and are in line with the results of He et al. (2020).

It is well known that N application and SOM significantly influence 1,000-grain weight and grain yield of wheat (Wang et al., 2018). Yan et al. (2019) showed that an increased fertilization rate of N, phosphorus, and potassium contributed to an increase in the duration of grain filling in winter wheat and obtained the yields ranging from 6,700 to 9,400 kg ha^−1^. Lv et al. (2017) reported that with yield levels ranging from 7,000 to 8,500 kg ha^−1^, potassium foliage application significantly increased grain weight by increasing the grain filling rate, whereas phosphorus foliage application relieved wheat senescence and increased D, leading to an increase in grain weight. In this study, mean soil nutrients in 0–40 cm soil layer during whole wheat growth stages were positively related to the 1,000-grain weight (Table 5). Positive correlations among grain filling characteristic parameters (i.e., W_max_, V_max_, and V_mean_), SOD and SP, and negative relationships between D and MDA (Table 6) indicated that alleviating flag-leaf senescence contributed to the improvement in the rate and duration of grain filling, and delayed leaf senescence positively contributes to prolonging source activity to grain filling and increased crop yield (Gelang et al., 2000; Derkx et al., 2012). Moreover, leaf photosynthesis is another contributor to the improvement of grain filling. Positive relationships were observed among photosynthetic characteristics and grain filling characteristic parameters (Table 6), suggesting that elevated leaf photosynthesis characteristics in HF contributed to the increase in grain filling rate.

Potassium fertilization and/or straw return alleviated soil potassium depletion and increased soil potassium fertility, thus leading to the increases in crop yields (Zhao et al., 2014). Increased plant-available soil N and phosphorus by maize straw mulch combined with no-tillage obtained the highest yields of 7,477 kg ha^−1^, leading to 16.7% increase in grain yield as compared to conventional tillage (Yang et al., 2020). Compared to the inorganic fertilizer treatment, long-term manure application (with grain yield levels up to 1,700 kg ha^−1^) increased the stock of soil organic carbon and TN, leading to the increase in crop yields (Qaswar et al., 2022). Schmitz and Ransom (2021) indicated that spike number, grain number and 1,000-grain weight are closely related to wheat yield in both high (>5,000 kg ha^−1^) and low (>5,000 kg ha^−1^) yield environments. In most of the high-yield fields, SOM, hydrolyzable N, AP, and AK were higher than in the mid-low-yield field, and effective spike number was the main yield component in the high-yield wheat field, followed by 1,000-grain weight (Peng et al., 2016). Comparable results were observed in the present study, and mean soil nutrients in 0–40 cm soil layer during whole wheat growth stages had positive relationships with spike number, 1,000-grain weight, and grain yield (Table 5). Yield advantage in HF came from the increased spike number and 1,000-grain weight advantage, leading to the yield gaps of 1,664 and 2,223 kg ha^−1^ compared to those seen for MF (Table 4). The decrease in spike number per m^2^ and grain number in 2017/18 resulted in a lower grain yield compared to 2018/19 (Table 4). This was due to the monthly precipitation fluctuations, and more rain occurred from jointing to maturity the following year (Figure 1). The guaranteed more water supply during the reproductive and grain filling stages contributed to the improvements of the yield formation (Mu et al., 2021).

## Conclusion

HF, with grain yield levels up to 11,000 kg ha^−1^, had higher soil nutrients than those for MF during wheat growth stages, providing sufficient nutrient supply during the wheat tillering period. Compared to MF, HF improved tiller production, including spike formation rate, resulting in an increased spike number. Furthermore, improvements in grain filling duration and grain filling rate in HF led to the higher 1,000-grain weight. These improvements were also due to the increased canopy light interception and leaf photosynthesis characteristics after anthesis, and alleviated leaf senescence in HF. These results suggest that increasing the spike formation rate, maximizing light interception, and increasing and extending the leaf photosynthesis contributed to narrowing the yield gap between high and middle soil nutrients fields.

## Data availability statement

The raw data supporting the conclusions of this article will be made available by the authors, without undue reservation.

## Author contributions

YS and ZY conceived and design the study and revised the manuscript. XZ and FY performed the experiments. XZ analyzed the data and wrote the manuscript. All authors contributed to the article and approved the submitted version.

## Funding

This study was supported by the National Natural Science Foundation of China (nos. 32172114 and 31771715) and the China Agriculture Research System of MOF and MARA (no. CARS-03).

## Conflict of interest

The authors declare that they have no known competing financial interests or personal relationships that could have appeared to influence the work reported in this paper.

## Publisher’s note

All claims expressed in this article are solely those of the authors and do not necessarily represent those of their affiliated organizations, or those of the publisher, the editors and the reviewers. Any product that may be evaluated in this article, or claim that may be made by its manufacturer, is not guaranteed or endorsed by the publisher.

## Supplementary material

The Supplementary materials for this article can be found online at: https://www.frontiersin.org/articles/10.3389/fpls.2022.932821/full#supplementary-material

## Footnotes

^1^ https://szb.farmer.com.cn/2019/20190807/20190807_001/20190807_001_5.htm

^2^ https://3g.163.com/dy/article/FGEV87NM0530WJTO.html
