# Supplementary material for: Grain-filling characteristics and yield formation of wheat in two different soil fertility fields in the Huang–Huai–Hai Plain
Source: Front Plant Sci. 2022 Jul 26;13:932821. doi: 10.3389/fpls.2022.932821 (PMC9364837; doi:10.3389/fpls.2022.932821)
Supplement: Supplementary file 1 [file Data_Sheet_1.pdf]

Supplementary Material for

Grain-filling characteristics and yield formation of wheat in two different soil fertility fields in the Huang–Huai–Hai Plain

Xuejiao Zheng, Zhenwen Yu, Fengxin Yu, Yu Shi*

This file includes:

Table S1.

Soil texture and particle size distribution before sowing at the experimental site in 2018/19 growing season.

Soil layers	High soil fertility field				Middle soil fertility field			
	Soil texture	Particle size distribution (%)			Soil texture	Particle size distribution (%)		
(cm)		Sand	Silt	Clay		Sand	Silt	Clay
		2.00-0.05 mm	0.05-0.002 mm	<0.002 mm		2.00-0.05 mm	0.05-0.002 mm	<0.002 mm
0-20	Sandy clay loam	65.97	9.30	24.73	Sandy clay loam	65.63	11.47	22.91
20-40	Sandy clay loam	66.64	10.30	23.06	Sandy clay loam	65.73	11.70	22.57
40-60	Sandy loam	63.97	17.13	18.90	Sandy loam	63.80	16.80	19.40
60-80	Sandy loam	64.66	18.13	17.21	Sandy loam	64.64	18.47	16.90
80-100	Sandy loam	61.94	24.70	13.36	Sandy loam	66.65	19.47	13.89
100-120	Sandy loam	61.19	26.30	12.51	Sandy loam	67.20	20.47	12.33
120-140	Sandy loam	62.55	23.47	13.98	Sandy loam	66.73	21.30	11.97
140-160	Sandy loam	63.40	21.97	14.63	Sandy loam	66.38	20.63	12.98
160-180	Sandy loam	74.35	11.13	14.52	Sandy loam	75.25	10.80	13.95
180-200	Sandy loam	76.86	8.33	14.81	Sandy loam	76.88	8.47	14.65

Table S2

Soil bulk density and filed capacity before sowing at the experimental site in 2017/18 and 2018/19 growing seasons.

Year	Index	Treatment	Soil layers (cm)									
			0-20	20-40	40-60	60-80	80-100	100-120	120-140	140-160	160-180	180-200
2017/18	Bulk density	HF	1.42b	1.58b	1.56a	1.59a	1.62a	1.63a	1.64a	1.63a	1.64a	1.64a
	($g\ cm^{-3}$)	MF	1.50a	1.64a	1.59a	1.61a	1.64a	1.65a	1.64a	1.65a	1.66a	1.65a
	Field capacity	HF	27.25a	23.31a	26.09a	24.75a	23.77a	23.40a	23.80a	23.74a	23.66a	23.61a
	(%)	MF	24.47b	21.92b	24.98a	24.09a	23.86a	22.69a	23.41a	23.04a	22.86a	23.30a
2018/19	Bulk density	HF	1.41b	1.56b	1.54a	1.57a	1.63a	1.64a	1.64a	1.65a	1.64a	1.65a
	($g\ cm^{-3}$)	MF	1.55a	1.63a	1.55a	1.59a	1.64a	1.65a	1.67a	1.65a	1.66a	1.66a
	Field capacity	HF	29.54a	25.44a	27.69a	25.35a	24.58a	24.22a	23.54a	23.64a	23.71a	23.08a
	(%)	MF	24.89b	22.08b	25.48b	24.96a	24.27a	24.11a	23.04a	22.75a	23.44a	23.03a

HF, high soil fertility field and MF, middle soil fertility field. Values followed by a different letter are significantly different (LSD test, $p < 0.05$) within the treatments in each year.

Table S3

The growth curve equations of grain filling process of each treatment with three replicates in 2017/18 and 2018/19 growing seasons.

Year	Treatment	Growth curve equation	Correlation coefficient
2017/18	HF	$y=46.6109/(1+45.7973e^{-0.2105x})$	0.9980
		$y=46.8455/(1+46.4878e^{-0.2097x})$	0.9988
		$y=46.5626/(1+44.1827e^{-0.2094x})$	0.9986
	MF	$y=43.2331/(1+49.2343e^{-0.2169x})$	0.9975
		$y=43.1698/(1+48.1446e^{-0.2160x})$	0.9978
		$y=42.8801/(1+48.3429e^{-0.2193x})$	0.9976
2018/19	HF	$y=47.0175/(1+47.6692e^{-0.2165x})$	0.9993
		$y=47.2094/(1+44.3558e^{-0.2111x})$	0.9995
		$y=47.0822/(1+44.8246e^{-0.2114x})$	0.9994
	MF	$y=42.9267/(1+48.4459e^{-0.2208x})$	0.9988
		$y=42.9954/(1+45.7597e^{-0.2198x})$	0.9989
		$y=43.0892/(1+48.1431e^{-0.2217x})$	0.9990

HF, high soil fertility field and MF, middle soil fertility field.
